# Yeast Probiotics Shape the Gut Microbiome and Improve the Health of Early-Weaned Piglets

**DOI:** 10.3389/fmicb.2018.02011

**Published:** 2018-08-23

**Authors:** Jinqiang Xu, Yuhui Li, Zhiqiang Yang, Chunhui Li, Hongyan Liang, Zuowei Wu, Wanxia Pu

**Affiliations:** ^1^Key Laboratory of Veterinary Pharmaceutical Development, Ministry of Agriculture, Key Laboratory of New Animal Drug Project of Gansu Province, Lanzhou Institute of Husbandry and Pharmaceutical Science, Chinese Academy of Agricultural Sciences, Lanzhou, China; ^2^Department of Veterinary Microbiology and Preventive Medicine, College of Veterinary Medicine, Iowa State University, Ames, IA, United States

**Keywords:** yeast probiotics, early-weaned piglets, gut microbiota, 16S rRNA, animal performance

## Abstract

Weaning is one of the most stressful challenges in the pig’s life, which contributes to dysfunctions of intestinal and immune system, disrupts the gut microbial ecosystem, and therefore compromises the growth performance and health of piglets. To mitigate the negative impact of the stress on early-weaned piglets, effective measures are needed to promote gut health. Toward this end, we tamed a *Saccharomyces cerevisiae* strain and developed a probiotic Duan-Nai-An, which is a yeast culture of the tamed *S. cerevisiae* on egg white. In this study, we tested the performance of Duan-Nai-An on growth and health of early-weaned piglets and analyzed its impact on fecal microbiota. The results showed that Duan-Nai-An significantly improved weight gain and feed intake, and reduced diarrhea and death of early-weaned piglets. Analysis of the gut microbiota showed that the bacterial community was shaped by Duan-Nai-An and maintained as a relatively stable structure, represented by a higher core OTU number and lower unweighted UniFrac distances across the early weaned period. However, fungal community was not significantly shaped by the yeast probiotics. Notably, 13 bacterial genera were found to be associated with Duan-Nai-An feeding, including *Enterococcus, Succinivibrio, Ruminococcus, Sharpea, Desulfovibrio, RFN20, Sphaerochaeta, Peptococcus, Anaeroplasma*, and four other undefined genera. These findings suggest that Duan-Nai-An has the potential to be used as a feed supplement in swine production.

## Introduction

The mammalian intestine is home to microorganisms, where trillions of microbes form a complex and dynamic ecosystem. The gastrointestinal microorganisms make great contributions to the host health and the digestive efficiency of nutrients, but they are also strongly affected by the host’s diet intake (Amato et al., 2013). Dysbiosis of gastrointestinal microflora may often result in dysfunction of intestinal microbiota and increased susceptibility to pathogens (Costello et al., 2012). The microorganisms in mammalian intestine play crucial roles in nutrient digestion and absorption (Backhed et al., 2015), the development of host immune and defense systems (Ivanov et al., 2009), the differentiation of intestinal epithelium (Sommer and Backhed, 2013), and the maintenance of intestinal mucosal barrier (Garrett et al., 2010).

In the swine industry, piglets are weaned early to meet the production goals, before a stable microbial population is built up and the immune system is fully developed. Weaning is one of the most stressful challenges pigs meet in their lives. Weaning can induce dysfunctions of the intestinal and immune system, consequently compromises the health, growth, and feed intake of piglets, especially during the 1st week after weaning (Campbell et al., 2013). The event can even further disrupt the gut microbial ecosystem and increase susceptibility to bacterial post-weaning diarrhea, causing substantial morbidity and mortality (Campbell et al., 2013).

Low levels of antibiotics (sub-therapeutic) in feed have been widely used to increase growth rates and prevent the bacterial post-weaning diarrhea for weaned pigs (van der Fels-Klerx et al., 2011; Heo et al., 2013). However, routine use of antibiotics in farm animals has significantly contributed to the increasing emergence of multi-drug resistant pathogens, incurring a major health concern in both animals and human (Landers et al., 2012). Moreover, in spite of the fact that sub-therapeutic antibiotics have demonstrated to improve growth performance, prolonged use may enrich the populations of potential pathogenic bacteria, such as *Salmonella, Escherichia coli*, and *Shigella* spp., and make them to remain long after the initial antibiotic treatment, exerting long-term negative effects on the host (Janczyk et al., 2007; Schokker et al., 2014).

Probiotics are proposed as alternatives to sub-therapeutic antibiotics in the livestock industry. It has been well-documented that yeast and yeast products are applicable as probiotics (Mathew et al., 1998; van Heugten et al., 2003; van der Peet-Schwering et al., 2007; Broadway et al., 2015). Dietary supplementation of yeast cultures and yeast products has been paid increasing attention for improving immune function and intestinal development in weaned piglets (Broadway et al., 2015), but their beneficial effects are as yet vague. In many studies, dietary supplementation of live yeast, yeast cultures or yeast cell wall products were shown to have positive effects on performance and health in weaned piglets by mitigating negative effects associated with stress and disease (Mathew et al., 1998; White et al., 2002; van Heugten et al., 2003; Rozeboom et al., 2005; Li et al., 2006). But in other studies, no beneficial effects were observed (Veum and Bowman, 1973; White et al., 2002; van Heugten et al., 2003). The administered form of probiotic yeast is also an important consideration. For instance, administration of liquid fermented yeast was more effective than dried yeast in animal performance improvement (Jiang et al., 2015). The liquid fermented diets were functional in maintaining the intestinal integrity of piglets during post-weaning periods, thereby diminishing post-weaning diarrheal symptoms (Upadrasta et al., 2013; Jiang et al., 2015).

*Saccharomyces cerevisiae* fermented egg white, known as active egg white product (AEWP) (Araki et al., 1992), has been reported to improve macrophage functions in mice (Araki et al., 1992) and to promote activation of neutrophilic functions in piglets (Araki et al., 1993) and calves (Nakagawa et al., 1993) by oral administration, contributing to host resistance to infections. In order to improve the growth performance of early-weaned piglets, we tamed *S. cerevisiae* strain S288c to grow well in egg white, and developed a yeast probiotic by fermenting egg white, which is named Duan-Nai-An in China. Duan-Nai-An was a product of live yeast and active egg white combination. In this study, Duan-Nai-An and another probiotic that was prepared as original *S. cerevisiae* S288c fermented malt were evaluated for their effect on the growth performance in early-weaned piglets during the first 10 days after weaning that is the most stressful period, and the modifying effects of the products on gut microbiota were analyzed by 16S rDNA and ITS sequencing for bacterial and fungal communities, respectively.

## Results

### Effect of Yeast Probiotics on Clinical Outcomes

**FIGURE 1:**
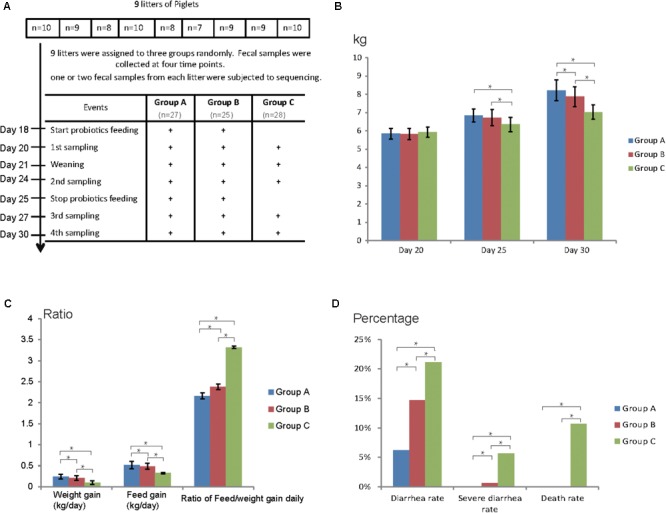
Experimental design and clinical data of piglets. **(A)** Experimental design, and workflow of yeast probiotics feeding and fecal sample collection. Group A was fed with probiotics Duan-Nai-An, Group B was fed with *Saccharomyces cerevisiae* fermented malt, and Group C was used as blank control without feeding any probiotics. **(B)** Weight of piglets from each group during the experimental window. **(C)** Weight gain, feed intake, and the ratio of feed/weight conversion per day of each group. **(D)** Diarrhea rate, severe diarrhea rate, and death rate of each group. Significant differences (*P* < 0.05) between the groups were indicated by stars.

Nine litters of hybrid piglets (Yorkshire, Landrace, and Duroc) were chosen for this study and all the piglets were raised under the same conditions in a commercial farm (**Figure 1A**). No antibiotics were applied for all the piglets. Nine litters were assigned to three groups randomly: Group A, Group B, and Group C, comprising of 27, 25, and 28 piglets (3 litters each), respectively. Group A was fed Duan-Nai-An, Group B was fed original *S. cerevisiae* fermented malt, Group C was fed basal diet, respectively, for 7 days (**Figure 1A**). Fecal samples were collected at the age of days 20, 24, 27, and 30 and five fecal samples (one or two from each litter) for each group at each time point were subjected to 16S rDNA and ITS sequencing. During the experimental period, yeast probiotics were found to significantly improve the health of weaned pigs, indicated by increased weight gain and feed intake as well as reduced diarrhea and death (**Figures 1B–D**). At days 25 and 30, the weight of piglets fed with yeast probiotics (Group A and Group B) was significantly higher (*P* < 0.05) than that in control Group C (**Figure 1B**). Notably, the weight of Group A was significantly higher (*P* < 0.05) than that in Group B at day 30. Daily weight and feed gain were significantly higher in Groups A and B compared to Group C, and the rate of daily feed/weight was significantly lower (*P* < 0.05) in Groups A and B (**Figure 1C**). Compared to Group B, Group A gained even more weight and feed intake daily, and had lower rate of daily feed/weight. Diarrhea rate, severe diarrhea rate, and death rate of Group A and Group B were significantly lower (*P* < 0.05) than those of Group C (**Figure 1D**). Again, Group A had a lower diarrhea rate (*P* < 0.05) than Group B. Together, the results showed that yeast probiotics significantly improved the health of weaned pigs and Duan-Nai-An was more effective than the original *S. cerevisiae* fermented malt.

### Yeast Probiotics Shaped the Bacterial Community of the Gut Microbiota

**FIGURE 2:**
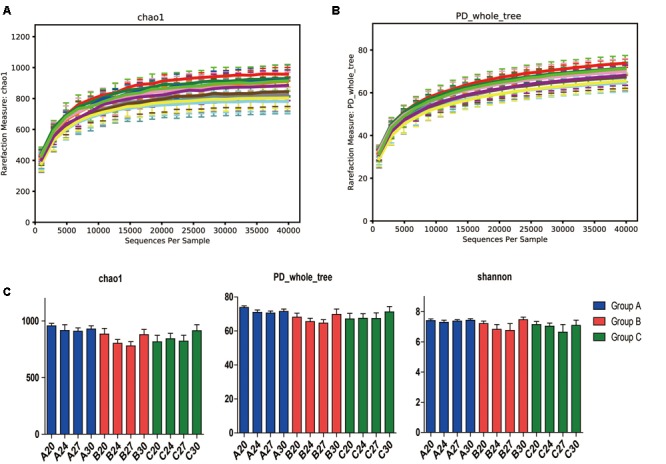
Species richness, phylogenetic diversity and evenness of fecal microbiota measured by 16S rDNA sequencing. Day-wise averages of the species richness and phylogenetic diversity of the individual samples at the age of days 20, 24, 27, and 30 for Groups A, B, and C were employed to prepare the figures, respectively. Rarefaction curves on the species richness (Chao1) and diversity (PD whole tree) are shown in **(A,B)**, respectively. Richness, diversity and evenness are shown in **(C)**. No significant differences (*p* < 0.05) were observed day-wisely between any of the samples for each day within groups. Data are shown as mean ± SEM.

**FIGURE 3:**
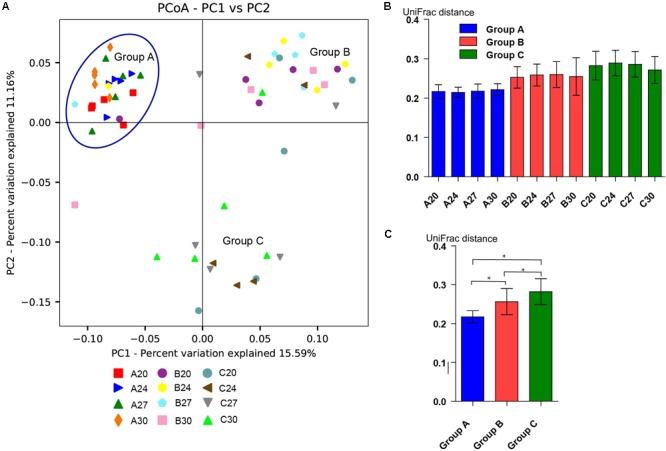
Beta diversity of the fecal bacterial community from days 20, 24, 27, and 30 in each group. **(A)** Principal coordinate analysis based on unweighted UniFrac metrics indicates that gut bacterial community are affected by yeast probiotics. The close clustering of the samples from each sampling day in Group A demonstrates the high phylogenetic similarities of their microbiota. The samples in Groups B and C are independent clusters, indicating that they are phylogenetically different groups. But samples in Groups B and C are not as closely related as those in Group A. Four samples in Group B clustered with Group A and eight samples in Group C clustered with Group B. **(B)** The day-wise averages of UniFrac distance for each group during the whole experiment period. No significant difference (*p* < 0.05) were observed between the samples from different days within groups. **(C)** The average UniFrac distance for all the samples in different piglet groups. Data are shown as mean ± SEM. Significant differences (*P* < 0.05) between the groups were indicated by stars.

To investigate the gut microbiota in the piglets, fecal samples were collected at the age of 20, 24, 27, and 30 days and 16S rDNA was sequenced. In total, we obtained 3,336,146 high-quality sequences of the V4 region in 60 fecal samples after quality control. The average high-quality sequences generated per sample were 55,602 for the bacterial populations. Rarefaction curves based on the alpha diversity metrics (Chao1 and PD whole tree indices) demonstrated that the sequencing depth were enough to capture the bacterial diversity in the feces of piglets (**Figures 2A,B**). Based on 97% sequence similarity, all the sequences of V4 region were clustered into 1681 bacterial OTUs. Richness, phylogenetic diversity, and evenness of the gut microbiota were then evaluated for each sample by the Chao1, PD whole tree, and Shannon indices, respectively (**Figure 2C**). Slight fluctuations were observed between samples from different days within groups and between groups, but the differences were not significant statistically (*P* > 0.05). Interestingly, no significant changes were observed between pre-weaning (day 20) and post-weaning samples (days 24, 27, and 30). To determine the phylogenetic variations with ages of piglets and probiotics, unweighted UniFrac distances were measured. PCoA analysis of bacterial OTUs based on the unweighted UniFrac distance metrics revealed that the samples clustered together according to probiotics feeding, but independent of ages (**Figure 3A**). However, only the samples of group A were clustered closely together, indicating that their bacterial community structures were highly similar and stable. The samples in group B and group C were loosely clustered. Four samples of Group B scattered among Group A, and 8 samples of Group C scattered among Group B. The UniFrac distances of the piglets within groups were similar, and no significant differences (*P* > 0.05) were observed between days (**Figure 3B**), but the distances were significantly lower in Group A and increased in Groups B and C (**Figure 3C**), suggesting increasing variations of the fecal bacterial community in the piglets fed with original *S. cerevisiae* fermented malt and without probiotics. These results suggest that yeast probiotics shaped the bacterial community of weaned piglets, and Duan-Nai-An had more impact on maintaining a stable structure of bacterial community.

### The Overall Bacterial Community Structure

**FIGURE 4:**
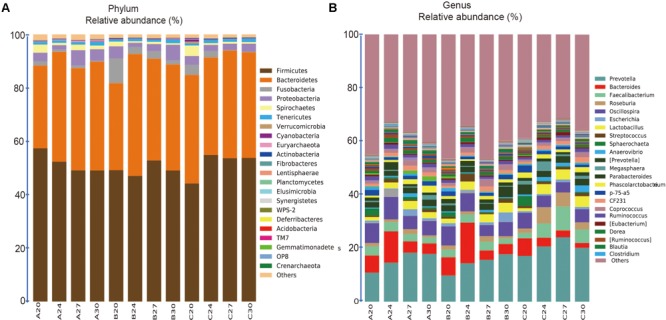
Stacked bar plots showing average percentage of bacterial populations in pig feces over time, from left to right, at days 20, 24, 27, and 30, for Groups A, B, and C, respectively. **(A)** Fecal bacterial composition evaluated at the phylum levels (23 taxa). **(B)** Fecal bacterial composition evaluated at the genus levels (175 taxa), but only the names of the top 23 abundant taxa (over 0.5% of the total sequences) were listed along with the plot.

The 1681 OTUs across all the 60 samples were classified into 22, 44, 77, 118, 176, 73 known taxa at Phylum, Class, Order, Family, Genus, Species levels, respectively. The 22 known phyla accounted for 98.6% of the total sequences, and the 176 known genera accounted for 61.7% of the total sequences. At the phylum level (**Figure 4A**), 6 out of 22 taxa were each comprised of over 1% of the total sequences (Firmicutes, Bacteroidetes, Fusobacteria, Proteobacteria, Spirochaetes, and Tenericutes), but Firmicutes and Bacteroidetes were the most dominant, accounting for 51.05 and 38.72% of the total sequences, respectively. Interestingly, the relative abundance of Firmicutes was declining along with aging within Group A, but increased within Group C and fluctuated within Group B. The second dominant phylum Bacteroidetes increased in Groups A and B, but not significantly in Group C. At the genus level (**Figure 4B**), 23 genera each contained more than 0.5% of the total sequences, accounting for 55.9% of the total sequences, and 38.3% sequences could not be assigned to known genus. *Prevotella* in phylum Bacteroidetes was the most abundant genus, accounting for 16.8% of the total sequences, whose relative abundance was increasing along with the age within all the three groups.

### Differences in Bacterial Communities Associated With the Yeast Probiotics

**FIGURE 5:**
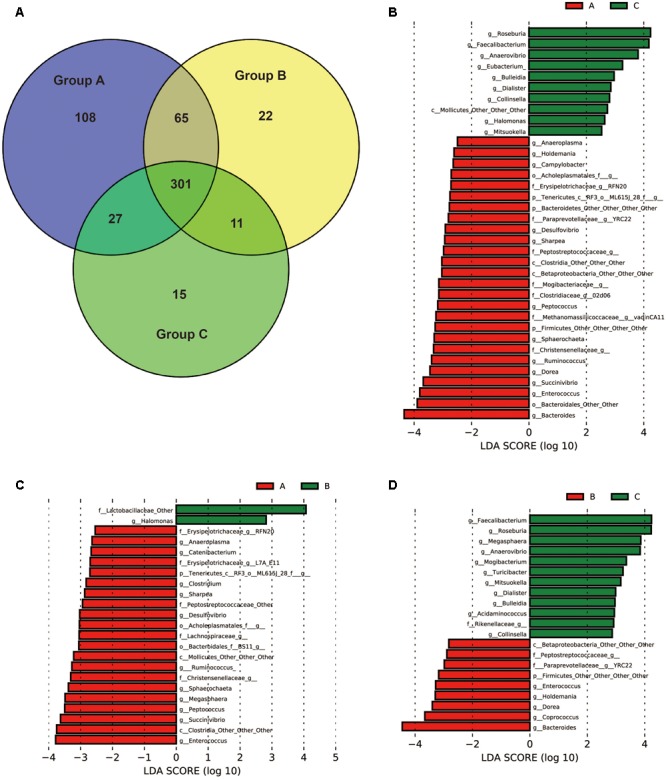
Bacterial groups affected by yeast probiotics. **(A)** Shared and unique core OTUs in the gut microbiota of Groups A, B, and C. To evaluate the number of unique and shared OTUs, a core microbiota (OTUs that were present in 100% of the samples in each group) was computed. VENNY 2.0.263 was used to identify the unique and shared OTUs in the different study groups. The Venn diagram shows the numbers of shared and unique core OTUs in the microbiota of each group. **(B–D)** Bacterial taxa (core OTUs of Group A) at the genus level significantly differentiated identified by linear discriminant analysis coupled with effect size (LEfSe) using the default parameters between Groups A and C, Groups A and B, Groups B and C, respectively.

To identify the bacterial taxa associated with probiotic feeding, core OUTs of each group were compared and LEfSe [Linear Discriminant Analysis (LDA) Effect Size] analysis was performed to detect the biomarkers. Pre-weaning samples (day 20) in each group were excluded for the analysis in order to specifically identify the taxa associated with health improvement by probiotics for weaned piglets. The shared core OTUs within groups for post-weaning samples (days 24, 27, and 30) were counted on the OTUs present in 100% of the samples of a particular group (15 out of 15 fecal samples). 501 core OTUs were identified in Group A, 399 core OTUs were identified in Group B, and 354 core OTUs were identified in Group C (**Figure 5A**). The numbers of core OTUs of each group were consistent with UniFrac distances as shown in **Figure 3**. The samples within Group A were closely related and shared highest number of OTUs, and less core OTUs were shared in loosely related samples in Group B and Group C. Core OTUs of Group A were then subjected to LEfSe analysis at the genus level in order to detect the biomarkers. The results showed that 26 taxa were significantly more abundant in Group A than in Group C (**Figure 5B**), 21 taxa were significantly more abundant in Group A than in Group B (**Figure 5C**), and 9 taxa were significantly more abundant in Group B than in Group C (**Figure 5D**), suggesting that they may be associated with probiotics feeding. Interestingly eight genera were more abundant in both Groups A and B than in Group C, which were *YRC22, Enterococcus, Dorea, Bacteroides, Holdemania*, and three other undefined genera. Thirteen genera were more abundant in Group A than in both Groups B and C, which were *Enterococcus, Succinivibrio, Ruminococcus, Sharpea, Desulfovibrio, RFN20, Sphaerochaeta, Peptococcus, Anaeroplasma*, and four other undefined genera.

### Fungal Compositions in Gut Microbiota

To examine whether fungal populations in the gut microbiota were affected by probiotics feeding, fecal samples of the piglets above were further analyzed by ITS sequencing. Eight out of the 60 samples were dropped due to the poor quality of sequences. In total, we got 2,260,472 high-quality sequences of ITS1 region for 52 samples after quality control and 43,471 on average for each sample. Based on 97% sequence similarity, all the sequences of the ITS1 region were assigned into 2791 OTUs, but only 1105 OTUs got blast-hits in the OTU database. PCoA analysis showed that fungal OTUs did not cluster together either according to age of piglets or groups (**Supplementary Figure S1**), which indicated that the fungal population was phylogenetically diverse and was not significantly shaped by aging or probiotics feeding during the experimental period. The fungal population in the gut microbiota of the piglets was found to include four defined taxa by RDP classifier at the phylum level, which were Basidiomycota, Ascomycota, Chytridiomycota, and Zygomycota. Basidiomycota, representing more than 33.00% of total sequences in all the samples, was the most dominant phylum, independent of ages. A total of 245 genera were detected in fungal communities of piglets, but their relative abundances were highly variable between samples within groups and between groups (**Supplementary Figure S2A**). Among them, *Amanita, Loreleia, Kazachstania, Mortierella, Ramariopsis, Arthrographis, Neocallimastix*, and *Heterochaetella* were the top genera (median value), and *Amanita* was the dominant genus, accounting for 31.7% (median value) of the total sequences (**Supplementary Figure S2B**).

## Discussion

Yeast and its derivatives have long been proposed as probiotics or prebiotics in swine diets. The effect of yeast on the immune system and on enteric microbiota has been investigated in many studies (Broadway et al., 2015). However, reports in the literature may not be consistent. It has been thought that the characteristics of a particular yeast strain, the physiologic stage of the animals, or the environment in which they are raised, is likely the cause (Monroy-Salazar et al., 2012). Therefore, comparisons between experiments are difficult. The present study was conducted to investigate the effect of different preparations of yeast (*S. cerevisiae* S288c) on growth performance and gut microbiota in early-weaned (10 days of post-weaning) piglets. Our findings showed that the dietary supplementation with yeast cultures improved feed conversion ratio and health of early weaned piglets. Notably, yeast fermented egg white (Duan-Nai-An) was significantly more effective than yeast fermentation malt. Considering that the two probiotics were prepared using the same strain but different media, the fermented egg white may have greatly contributed to the performance of probiotics. The findings indicated that yeast probiotics effectively help piglets overcome the stress induced by weaning at the early production stage. However, as noticed in other studies (Jiang et al., 2015), the administered form of yeast probiotic is also important. Although it is impractical to administrate the yeast probiotics by oral gavage in pig production, the liquid format should be applied.

Bacterial communities between the yeast probiotics supplemented groups and control diet group at days 20, 24, 27, and 30 were compared. The species richness, diversity, and evenness of the bacterial communities did not show significant differences between groups, suggesting that alpha diversities were not affected significantly by the yeast probiotics during the experimental period in this study. No significant differences were observed to be related to aging and weaning either (piglets were weaned at day 21). It has been reported that alpha diversities of gut microbiota would increase with aging in pigs, but at a gradual manner (Upadrasta et al., 2013; Frese et al., 2015; Mach et al., 2015; Niu et al., 2015). Frese et al. (2015) showed that alpha diversities increased gradually from birth through 21 days, but plateaued after day 21 through day 42 and were not significantly changed by the weaning at day 21, which is consistent with our observations. But Niu et al. (2015) found that alpha diversities were still increasing after weaning when they examined the diversities at a longer interval of 30 days after weaning. Therefore, the short period of this study might not be enough to capture the significant changes of alpha diversities of gut microbiota with aging. However, significant differences were observed for bacterial community structure (Beta diversity) between groups, indicating that yeast probiotics shaped the bacterial community structure of gut microbiota in early-weaned piglets.

Consistent with previous studies on pigs (Lamendella et al., 2011; Kim et al., 2012; Looft et al., 2012), Firmicutes and Bacteroidetes were the two most dominant taxa at the phylum level and *Prevotella* was the most dominant taxa at the genus level in both yeast probiotics fed piglets and non-probiotics fed piglets. The relative abundance of genus *Prevotella* was increasing with aging in all the groups, but the relative abundance of the dominant phyla was different. Firmicutes was declining in Duan-Nai-An fed piglets but increasing in non-probiotics fed piglets, and fluctuated in original *S. cerevisiae* fermented malt fed piglets. Bacteroidetes increased in both yeast fed groups, but not significantly in group without probiotic feeding. In early-weaned piglets, other studies have shown that Firmicutes were most abundant in pre-weaning piglets, but shifted gradually to Bacteroidetes after weaning (Upadrasta et al., 2013; Pajarillo et al., 2014). The results in this study may suggest that Duan-Nai-An sped up the shift and adapted piglets more quickly to the weaned situation.

At the genus level, 8 taxa were significantly more abundant in the yeast probiotics fed piglets than in the non-probiotics fed piglets, and 13 taxa were significantly more abundant in Duan-Nai-An fed piglets than in both the original *S. cerevisiae* fermented malt fed piglets and non-probiotics fed piglets. Among them, interestingly, *Bacteroides* was more abundant in both yeast probiotics fed piglets. Previous reports have established *Bacteroides* species as some the early colonizers of pig gut (Poroyko et al., 2010; Lu et al., 2014; Mach et al., 2015), and considered them to be clinically important anaerobes in relation to gastrointestinal well-being (Pajarillo et al., 2014). Among the 13 significantly more abundant taxa in Duan-Nai-An fed piglets, *Enterococcus* strains have been commercialized as probiotics, and could prevent the colonization and stabilization of microbiota unfavorable to the host by competing for adhesion sites in the epithelial cells with pathogens (Araujo and Ferreira, 2013). *Succinivibrio* and *Ruminococci* are high-cellulose-degrading bacteria, playing a major role in helping to digest the complex carbohydrates (Flint, 2004; Graf et al., 2015). *Sharpea* is involved in the production of butyrate, a short-chain fatty acid (SCFA), by increasing lactate production as the substrate for *Megasphaera* to convert to butyrate (Kamke et al., 2016). SCFAs are the end products of fermentation of dietary fibers by intestinal microbiota, and have multiple beneficial effects on mammalian energy metabolism (den Besten et al., 2013). *Desulfovibrio* strains remove excess hydrogen generated during the fermentation of plant polysaccharides by intestinal microbiota, therefore remove the hydrogen inhibition on the fermentation process for continuous SCFA production (Bik et al., 2017). Although the roles of the other taxa are not clear, the known functions have indicated the taxa biomarkers may have inhibited the colonization of pathogens and improved the energy conversion of diets, and their close associations with yeast probiotics may suggest that they are important for growth performance and health improvement of piglets during the early weaned stage.

Few studies have investigated the gut fungal communities of pigs. In this study, we examined the fungal communities in gut microbiota of early-weaned piglets. The results showed that Basidiomycota, Ascomycota, Chytridiomycota, and Zygomycota were the main phyla, which are consistent with the main fungal phyla in human gut (Sam et al., 2017). Basidiomycota was the most dominant phylum. A total of 245 genera were detected in fungal communities of piglets. *Amanita* was the most dominant genus. However, at the genus level, a large variety of fungi were found in the human GI tract and there was no consensus yet on the ideal fungal community (Sam et al., 2017). As we saw in this study, no association was detected with yeast probiotics feeding and the fungal community was highly variable over time and even between individuals under the same treatment as well as untreated controls (**Supplementary Figures S1, S2**). Similar variations have also been observed for fungal communities in a study of mice, where the dominant fungal taxa varied in mice in different cages receiving the same treatment and gut mycobiome varied substantially over time in mice housed in the same animal facility and on a homogeneous diet (Dollive et al., 2013). Gut mycobiome was also reported to be highly variable in human between individuals as well as within individuals over time with the same controlled diets (Nash et al., 2017; Auchtung et al., 2018). In human, fungi has been considered transient members rather than true colonizers of the GI tract, and their presence in the gut is highly dynamic (Auchtung et al., 2018). Although we only examined the fungal communities for 10 days, it is tempting to speculate that fungi are just as transient members of the GI tract of pigs.

## Conclusion

Two yeast probiotics (named *S. cerevisiae* fermented egg white and original *S. cerevisiae* fermented malt) were tested in the present study in order to improve the growth performance and health of early-weaned piglets. The results showed that tamed *S. cerevisiae* fermented egg white (Duan-Nai-An) was effective in improving the health and growth of early-weaned piglets. The bacterial community of gut microbiome was significantly shaped by Duan-Nai-An. Duan-Nai-An was effective to maintain a stable bacterial community in gut microbiota for early-weaned piglets. Thirteen bacterial genera were found to be closely related to Duan-Nai-An feeding. However, fungal community of the gut microbiome was not shaped by yeast probiotics. The findings in this study suggest that Duan-Nai-An has the potential to be used as a supplement in swine industry.

## Materials and Methods

### Ethics

The experimental procedures performed in this study were approved by Animal Care and Use Committee of Lanzhou Institute of Husbandry and Pharmaceutical Science, The Chinese Academy of Agricultural Sciences.

### Yeast Probiotics Preparation

*Saccharomyces cerevisiae* S288c was used in this study to prepare the yeast synbiotics. Strain S288c was low pH tolerant (survival rate: 95–100% at pH 2–4 for 3 h) and bile salt tolerant (survival rate: 81–100% at 0.03–1% bile salt for 3 h). *S. cerevisiae* fermented malt was prepared as described previously (Li et al., 2014). The original *S. cerevisiae* S288c strain grew poorly under the presence of egg white powder. We tamed the strain by supplementing malt broth with 1% egg white powder at the beginning, then gradually up to 8%. After tamed for 1 year, S288c grew well in malt broth with 8% egg white powder. Viable yeast cells in both *S. cerevisiae* fermented malt and *S. cerevisiae* fermented malt plus 8% egg white (Duan-Nai-An) were about 2.0 × 10^8^ CFU/ml. The survivability test showed that the viable CFUs in both *S. cerevisiae* preparations did not change significantly after being kept at 4°C for 30 days.

### Animals and Sample Collection

All the piglets were from Tianzhu Tibetan Autonomous County Omnitec Farming Cooperatives in Gansu Province and fed under the same conditions. In total, 80 piglets in 9 litters were included in the study. The piglets were assigned into three groups. Group A was fed with basal diet and yeast probiotic Duan-Nai-An, Group B was fed with basal diet and original *S. cerevisiae* fermented malt, and Group C was used as blank control and fed with basal diet only. Yeast probiotics were fed 10 ml/day at the age of 18 days by oral gavage and continued 7 days until the age of 25 days. All the piglets were weaned at age of 21 days. Food intake was recoded daily, weight was recorded at day 20, 25, 30, and feed/weight gain ratio was calculated accordingly. Health evaluation (i.e., diarrhea rate, severe diarrhea rate, and death rate) were performed as described previously (Xie et al., 2017). Fecal samples were collected at the age of 20, 24, 27, and 30 days and frozen in liquid nitrogen immediately, and then stored at -80°C until genomic DNA extraction. Five fecal samples (one or two from each litter) in each group at each time point were applied for sequencing analysis in this study. The samples were named after the combination of groups, days, and animal numbers, for example A20-1, A24-1, A27-1, and A30-1 for Group A. In total, 60 fecal samples were included in this study.

### Microbial Genomic DNA Extraction and Amplification of 16S rDNA and ITS1 Regions

Total genomic DNA in the feces of piglets was extracted by CTAB method (Zhang et al., 2006). Bacterial 16S rDNA V4 region was amplified by primer pair 515F-806R, and fungal ITS1 region was amplified by primer pair ITS5-1737F and ITS2-2043R. All PCR reactions were carried out in a total volume of 30 μl reaction, including 15 l of Phusion High-Fidelity PCR MasterMix (Biolabs, New England), 0.2 mM of forward and reverse primers, and 10 ng templates DNA. Thermal cycling was consisted of an initial denaturation at 98°C for 1 min, 30 cycles of denaturation at 98°C for 10 s, annealing at 50°C for 30 s, and elongation at 72°C for 60 s. Finally the PCR reaction was held at 72°C for 5 min. PCR products were purified by GeneJET Gel Extraction Kit (Thermo Scientific) and subjected to sequencing.

### Library Preparation and Sequencing

Library preparation and sequencing were performed by Novogene (Beijing, China). Briefly, sequencing libraries were generated using TruSeq DNA PCR-free Library Prep Kit from Illumina (NEB, United States) following manufacturer’s protocol, and index codes were added. The library quality was assessed on the Qubit@ 2.0 Fluorometer (Thermo Scientific) and Agilent Bioanalyzer 2100 system. The library was sequenced on an Illumina HiSeq platform and 250 bp paired-end reads were generated. Reads data were deposited in the Sequence Read Archive (SRA) under accession SRP129888.

### Data Analysis

Raw reads was pre-processed to remove the adapters and low quality reads by the following procedures according to Qiime tags quality control process (Caporaso et al., 2010): (a) reads trimming, Raw reads were trimmed for the continuous low quality base (Q Score ≤ 19, length ≥ 3); (b) Reads length filter, reads shorter than 75% original length were filtered out. After quality control, chimera reads were removed by UCHIME (Edgar et al., 2011) and database searching (Haas et al., 2011). The overlapped paired-end clean reads were merged by Connecting Overlapped Pair-End (COPE, V1.2.1) (Liu et al., 2012). Bacterial operational taxonomic units (OTUs) were assigned at 97% sequence similarity by Mothur (v1.31.2) (Schloss et al., 2009). Bacterial OTU taxa were classified based on the Ribosomal Database Project (RDP) database (Cole et al., 2009). Fungal OTUs were assigned at 97% sequence similarity by USEARCH (v7.0.1090) (Edgar, 2013). Fungal OTU representative sequences were taxonomically classified using RDP Classifier v.2.2 based on the UNITE database (Abarenkov et al., 2010). Alpha diversity and Beta diversity were analyzed by QIIME (v1.80) (Caporaso et al., 2010). Alpha diversity analysis included Shannon index (abundance and evenness), Chao1 (richness) and PD_whole_tree (phylogenetic diversity). Beta diversity was measured by unweighted UniFrac metrics (phylogenetic beta diversity), and the distances were visualized by Principal Coordinate Analysis (PCoA). Mann–Whitney test was used for significance test of growth performance and Fisher’s exact test was used for significance test of health improvement. One-way ANOVA test was used for significance test of alpha diversity and beta diversity. All the statistics above were performed in GraphPad (Anderson et al., 2016), and *p*-value less than 0.05 was significant. Linear discriminant analysis coupled with effect size (LEfSe) was performed to identify the bacterial taxa differentially represented between groups at genus (Segata et al., 2011).

## Author Contributions

WP and ZY designed the research. WP, YL, HL, CL, and JX performed the research. ZW and JX analyzed the data. JX, ZW, and WP wrote the paper.

## Conflict of Interest Statement

The authors declare that the research was conducted in the absence of any commercial or financial relationships that could be construed as a potential conflict of interest.

## Supplementary Material

FIGURE S1 Beta diversity of the fecal fungal community from days 20, 24, 27, and 30 in each group. Principal coordinate analysis based on unweighted UniFrac metrics indicated that gut fungal community were not affected by yeast probiotics.

FIGURE S2 Thirteen top fungal genera (over 0.01% of the total sequences in relative abundance at median value across all the 52 samples) in the gut microbiota. **(A)** Area plot showed the relative abundance of each top genera across all the 52 samples. **(B)** Pie chart showed the median value of the relative abundance for each genus.
