# Supplementary figures and images for: Yeast Probiotics Shape the Gut Microbiome and Improve the Health of Early-Weaned Piglets
Source: Front Microbiol. 2018 Aug 23;9:2011. doi: 10.3389/fmicb.2018.02011 (PMC6119770; doi:10.3389/fmicb.2018.02011)

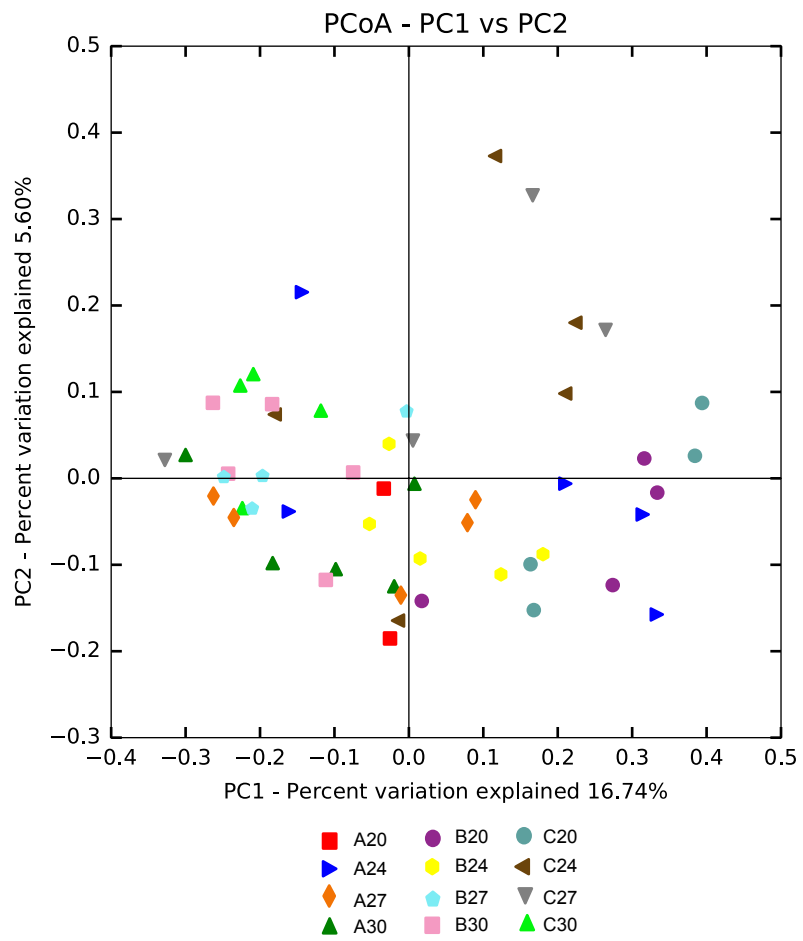

Supplement: FIGURE S1 — Beta diversity of the fecal fungal community from days 20, 24, 27, and 30 in each group. Principal coordinate analysis based on unweighted UniFrac metrics indicated that gut fungal community were not affected by yeast probiotics. [file Presentation_1.PDF]
