# Supplementary material for: Yeast Probiotics Shape the Gut Microbiome and Improve the Health of Early-Weaned Piglets
Source: Front Microbiol. 2018 Aug 23;9:2011. doi: 10.3389/fmicb.2018.02011 (PMC6119770; doi:10.3389/fmicb.2018.02011)
Supplement: FIGURE S2 — Thirteen top fungal genera (over 0.01% of the total sequences in relative abundance at median value across all the 52 samples) in the gut microbiota. (A) Area plot showed the relative abundance of each top genera across all the 52 samples. (B) Pie chart showed the median value of the relative abundance for each genus. [file Presentation_2.PDF]

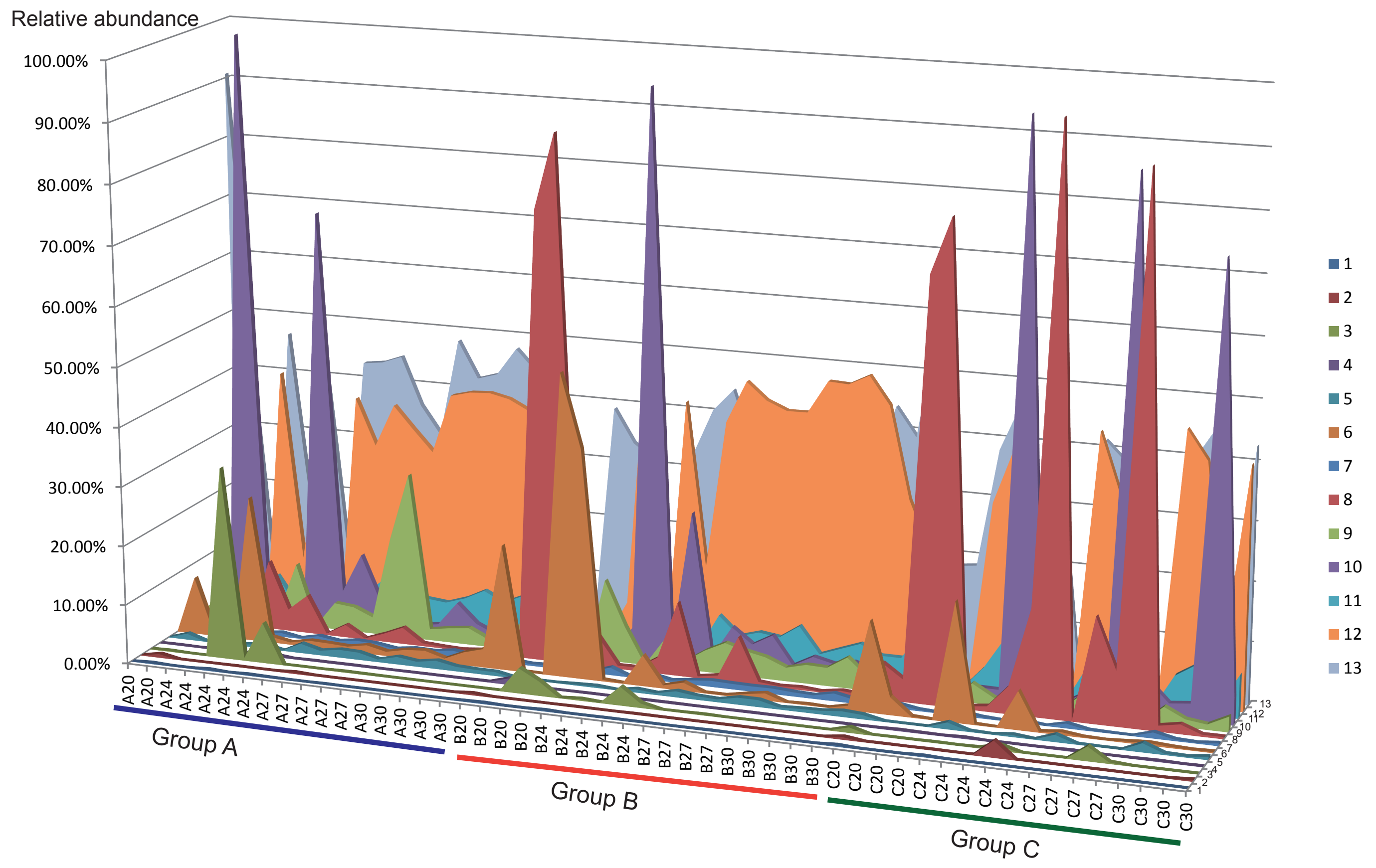

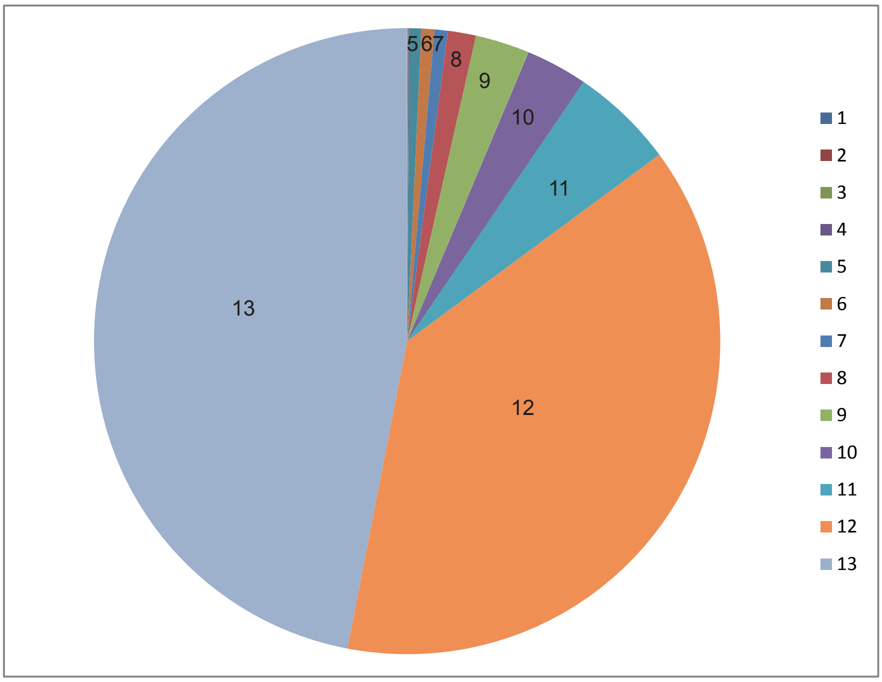

Median > 0.01% taxa

1.k__Fungi;p__Ascomycota;c__Sordariomycetes;o__unidentified;f__unidentified;g__unidentified;
2.k__Fungi;p__Basidiomycota;c__Agaricomycetes;o__Auriculariales;f__Incertae sedis;g__Heterochaetella;
3.k__Fungi;p__Chytridiomycota;c__Neocallimastigomycetes;o__Neocallimastigales;f__Neocallimastigaceae;g__Neocallimastix;
4.k__Fungi;p__Ascomycota;c__Dothideomycetes;o__Incertae sedis;f__Eremomycetaceae;g__Arthrographis;
5.k__Fungi;p__Basidiomycota;c__Agaricomycetes;o__Agaricales;f__Clavariaceae;g__Ramariopsis;
6.k__Fungi;p__Basidiomycota;c__unidentified;o__unidentified;f__unidentified;g__unidentified;
7.k__Fungi;p__Zygomycota;c__Incertae sedis;o__Mortierellales;f__Mortierellaceae;g__Mortierella;
8.Others
9.k__Fungi;p__Ascomycota;c__unidentified;o__unidentified;f__unidentified;g__unidentified;
10.k__Fungi;p__Ascomycota;c__Saccharomycetes;o__Saccharomycetales;f__Saccharomycetaceae;g__Kazachstania;
11.k__Fungi;p__Basidiomycota;c__Agaricomycetes;o__Incertae sedis;f__Incertae sedis;g__Loreleia;
12.k__Fungi;p__Basidiomycota;c__Agaricomycetes;o__Agaricales;f__Amanitaceae;g__Amanita;
13.k__Fungi;p__unidentified;c__unidentified;o__unidentified;f__unidentified;g__unidentified;
